# Clinical application of a modified predeposit autologous red blood cell apheresis in multistage spinal fusion: a single-center retrospective study

**DOI:** 10.3389/fmed.2023.1149093

**Published:** 2023-05-15

**Authors:** Xiao-Ping Xu, Wen-Jun Que, Ze-Bo Yu, Jie-Liang Shen, Zhen-Ming Hu, Xiao-Liang Yang, Jie Hao

**Affiliations:** ^1^The Department of Orthopedics, The First Affiliated Hospital of Chongqing Medical University, Chongqing, China; ^2^The Department of Blood Transfusion, The First Affiliated Hospital of Chongqing Medical University, Chongqing, China

**Keywords:** pre-deposit autologous RBC apheresis, multistage spinal fusion, length of stay, transfusion cost, post-operative infection

## Abstract

**Purpose:**

This study aimed to evaluate the efficacy and safety of predeposit autologous RBC apheresis (PARA) in patients undergoing multilevel spinal fusion surgery.

**Methods:**

A total of 112 patients from January 2020 to June 2022 were divided into two groups according to PARA: the PARA group (*n* = 51) and the control group (*n* = 61). The baseline characteristics of the patients, outcomes, transfusion cost, hospitalization cost, length of stay, complications, and changes in hemoglobin and hematocrit levels between the two groups were compared.

**Results:**

The baseline characteristics were similar in both groups. No significant differences were found in functional outcomes, including VAS score (*p* = 0.159), ODI score (*p* = 0.214), JOA score (*p* = 0.752), and SF-36 score (*p* = 0.188) between the PARA and control groups. The amount and rate of intraoperative and perioperative allogeneic RBC transfusion were significantly higher in the control group than in the PARA group (*p* < 0.001). The postoperative (9.04 ± 3.21 vs. 11.05 ± 3.84, *p* = 0.004) and total length of stay (15.78 ± 3.79 vs. 17.36 ± 4.08, *p* = 0.038) in the PARA group were significantly lower than those in the control group, respectively. Despite no difference in hospitalization cost (*p* = 0.737), the total blood transfusion cost in the PARA group was significantly lower, compared with the control group (*p* < 0.001). For safety evaluation, there were no significant differences in Hb and Hct levels between the two groups at admission, on postoperative day 1, and postoperative day 3, respectively (*p* > 0.05). Moreover, the number of postoperative infections in the PARA group was significantly lower than that in the control group (*p* = 0.038).

**Conclusion:**

PARA was a novel, safe, and highly efficient technique for mass autologous blood preparation in a quite short preparation time. This method could significantly reduce the amount of allogeneic blood transfusion and length of stay, which could provide a theoretical basis for following clinical practice about the technique.

## 1. Introduction

Perioperative allogeneic blood transfusion is often required in patients undergoing multilevel spinal fusion surgery, due to the complexity of the surgery and the high risk of intraoperative mass blood loss (1–3). Transfusion could solve the problems such as anemia and hypovolemia caused by bleeding profusely (4, 5). However, adverse blood transfusion reactions and lack of blood supply are still unavoidable. Compared with allogeneic transfusion, autologous transfusion can avoid the risks of allergic reactions, infectious disease transmission, and immune system inhibition, as well as save blood resources (6–8). Thus, in recent years, autologous blood transfusion has been gaining popularity gradually (9, 10). In particular, blood resources are in short supply during the COVID-19 pandemic, leading to the exhaustion of allogenic blood stored in hospitals (11).

Autologous blood transfusion is classified as predeposit autologous transfusion (PAT), salvage autologous transfusion (SAT), and acute normovolemic hemodilution (ANH) (9). SAT and ANH are widely used in preoperative blood preparation for a variety of surgeries, such as orthopedics, cardiothoracic, vascular surgery, gynecology, and obstetrics (12, 13). SAT, which could recycle the blood lost in the surgical field or body cavity, is a simple method to save allogeneic blood transfusion, but it is limited to intraoperative (14) procedures. ANH is used as a standard treatment for total hip arthroplasty in the United States; however, the operation must be performed in the operating room by anesthesiologists (15). PAT mostly adopts the step-volume method, which is simple to operate, but the blood collection volume is too small (up to 400 ml/week/person in China and 500 ml/time/person in European and American countries) to shorten the average hospital stay of patients with a large amount of blood preparation. This is the major reason for the steady marginalization of PAT in many countries recently (9, 16).

Therefore, in order to make up for the disadvantages of the aforementioned methods, a novel modified PAT, defined as predeposit autologous RBC apheresis (PARA), was performed by integrating PAT and ANH in this study. By using PARA collection once, 4–5 U of autologous concentrated RBC (100 ml = 1 U) could be provided for preoperative blood preparation within approximately 30 min. By exploring the safety and efficacy of PARA compared with allogeneic blood transfusion, this study could provide a new idea for perioperative patient blood management.

## 2. Materials and methods

### 2.1. Research design

We conducted a single-center, retrospective study that reviewed electronic medical records from subjects who underwent multilevel (≥3) spinal fusion surgery. The included patients received PARA or did not receive PARA in the Department of Orthopedics of our hospital from January 2020 to June 2022. The decision of whether to implement PARA before surgery was at the surgeon's discretion. Written informed consent was conventionally signed by all patients at admission. This study was approved by the Ethics Committee of the First Affiliated Hospital of Chongqing Medical University (Approval No: 2020-232).

### 2.2. Patients' data

In total, 129 patients who underwent multilevel (≥3) spinal fusion surgery were preliminarily enrolled in the study. Inclusion criteria were as follows: (1) Patients who were above 18 years of age in both sexes. (2) Patients who have American Society of Anesthesiology (ASA) anesthesia risk of I to IV. (3) Patients with a body mass index (BMI) of < 30 kg/m^2^ and ≥18.5 kg/m^2^. (4) Patients undergoing ≥3 levels of spine fusion surgery with a posterior midline approach. (5) Patients with preoperative hemoglobin ≥110 g/L and normal coagulation function (17). (6) Patients without anticoagulant therapy before surgery. Exclusion criteria were as follows: (1) multiple injuries; (2) tumor and infection beyond spine, or osteoporotic spinal condition as the reason for surgery; (3) preoperative hypertension≥160/100 mmHg; (4) arrhythmia; (5) mental disorder and (6) history of blood donation adverse reactions.

In all, 17 cases were excluded, including four cases of age >75 years old, eight cases of preoperative hemoglobin < 110 g/L, two cases of blood pressure higher than 160/100 mmHg before blood collection, one case of abandonment of surgery after autologous blood collection, and two cases of incomplete data. Finally, 112 patients were analyzed in this study. A total of 51 patients who received PARA were set as the experimental group, and 61 patients who received routine allogeneic blood preparation before surgery were set as the control group.

### 2.3. Surgical characteristics and interventions

All the patients enrolled were managed with spinal fusion surgery involving the thoracic and/or lumbar spine using a posterior midline approach. The surgical project was determined by consensus among the participating surgeons, which usually consists of posterior incision, posterior vertebral arches, and intervertebral disk decompression, with or without posterior spinal osteotomy, interbody fusion, and instrumentation with pedicle screws and wires of at least four vertebrae. In total, two ordinary-pressure drainage tubes were placed on both sides of the wound before closing the incision. The primary diagnoses of the participants included multisegmental intervertebral disk herniation, canal stenosis, degenerative scoliosis, idiopathic scoliosis, spondylolisthesis, or sagittal imbalance.

### 2.4. PARA protocol

Patients in the experimental group received PARA bedside 5 days before operation by NIGALE Blood Composition Separator (NGLXCF-3000) manufactured by Sichuan Biomedical Co., Ltd., Chengdu City, Sichuan Province, China. Before blood collection, the preoperative autologous RBC Storage Treatment Consent Form was signed. A single collection volume of 400 to 500 ml autologous RBC was evaluated by the blood bank staff using the clinician's advice according to the patient's hemoglobin level.

The whole process was performed by the blood bank staff in strict accordance with the standard operating procedures. For safety, an ECG monitor was carried out during the whole process. In brief, an appropriate peripheral vein was selected for puncture. After the startup process, the related program and parameters were set according to the instruction. Anticoagulant Citrate Dextrose Solution I (Sichuan NIGALE Biological Co., Ltd., China) was automatically added by the machine at a proportion of anticoagulant: blood = 1: 11. After initiating the blood collection preparation, venipuncture was performed with the 16G needle from the consumables after disinfection. The “blood collection” button was pressed to run the machine automatically. When 400 to 500 ml autologous RBC was collected, the remaining whole blood and plasma left in the machine were transfused back into the patient automatically. During the process, 250 ml of 5% glucose solution combined with 10% calcium gluconate 1 g and the same amount of normal saline was supplied by intravenous drip to avoid hypocalcemia caused by citrate and to maintain the balance of blood volume in and out. After collection, Anticoagulant Citrate Dextrose Solution I (Sichuan NIGALE Biological Co., Ltd., China) was added into autologous RBC at a ratio of 1:7 following the guideline (17), and then the RBC collection bag was closed by a heat machine (GIR-III, Suzhou Medical Equipment Factory Co., Ltd., Suzhou City, Jiangsu Province, China) and stored in a blood storage refrigerator (XC-240L, Zhongke Meiling Cryogenic Technology Co., Ltd., Hefei City, Anhui Province, China) at 4°C ± 2°C for 35 days (17). PARA should be discarded immediately by autoclaved sterilization if abnormal blood quality occurs, such as clotting, hemolysis, or blood bag damage. After PARA, 200 mg of iron sucrose through intravenous infusion was given to support the treatment. All patients in the PARA group underwent the intraoperative autologous blood transfusion. In the control group, 4–6 U allogeneic RBC application was submitted to the Department of Blood Transfusion by clinicians 1 day before the operation.

### 2.5. The formula for the amount of PARA collection

The PARA collection was performed based on the following formula. The actual blood collection volume was not more than 600 ml/case.

The amount of collection (ml) = weight (kg) ^*^ 7%^*^1000 (Hct _Beforethecollection_-Hct _targetvalue_)/0.75.

Note: 7%: Blood volume is equivalent to 7% of body weight.0.75: The hematocrit of concentrated autologous RBC in the collection bag was approximately 0.75 (0.7–0.8).Hct _target_ = 0.3 (based on AHN) (18).

### 2.6. Outcomes

The following demographic data were first collected: age, gender, BMI, the ASA physical status, perioperative coagulation and hematologic parameters, and the number of fusion levels. The primary outcome measures were the functional outcomes, including the visual analog scale (VAS) score, Oswestry Disability Index (ODI) score, Japanese Orthopedic Association (JOA) score, 36-term Short-Form Health Status Survey score (SF-36), and a total number of transfusion units and cost required during the perioperative period. The threshold for transfusion of blood products was hemoglobin ≤ 7 mg/dl. The secondary outcome measures including the hemoglobin (Hb) level and hematocrit (Hct) before blood collection, 1 day after blood collection, 1 day after surgery, and 3 days after surgery were collected in the experimental group. Meanwhile, the blood collection volume and adverse reactions (allergic reactions, non-hemolytic febrile reactions, acute hemolytic reactions, delayed hemolytic reactions, transfusion-related acute lung injury, and transfusion-related graft-vs.-host disease) of blood collection were also recorded to determine the safety of PARA.

### 2.7. Statistical Analysis

Statistical analysis was performed using SPSS version 22.0 (IBM Corp, Armonk, NY, USA). The continuous variables are presented as means ± standard deviations for normally distributed data, and Student's *t*-test was used to compare the differences in patient characteristics. Non-normally distributed variables were presented as medians (interquartile ranges, IQRs), and the differences between groups were tested using the Mann–Whitney U-test. The categorical variables were presented as frequencies (percentages) and compared using the chi-square test. Statistical significance was set at *P* < 0.05.

## 3. Results

### 3.1. Patient demographics

**Figure 1:**
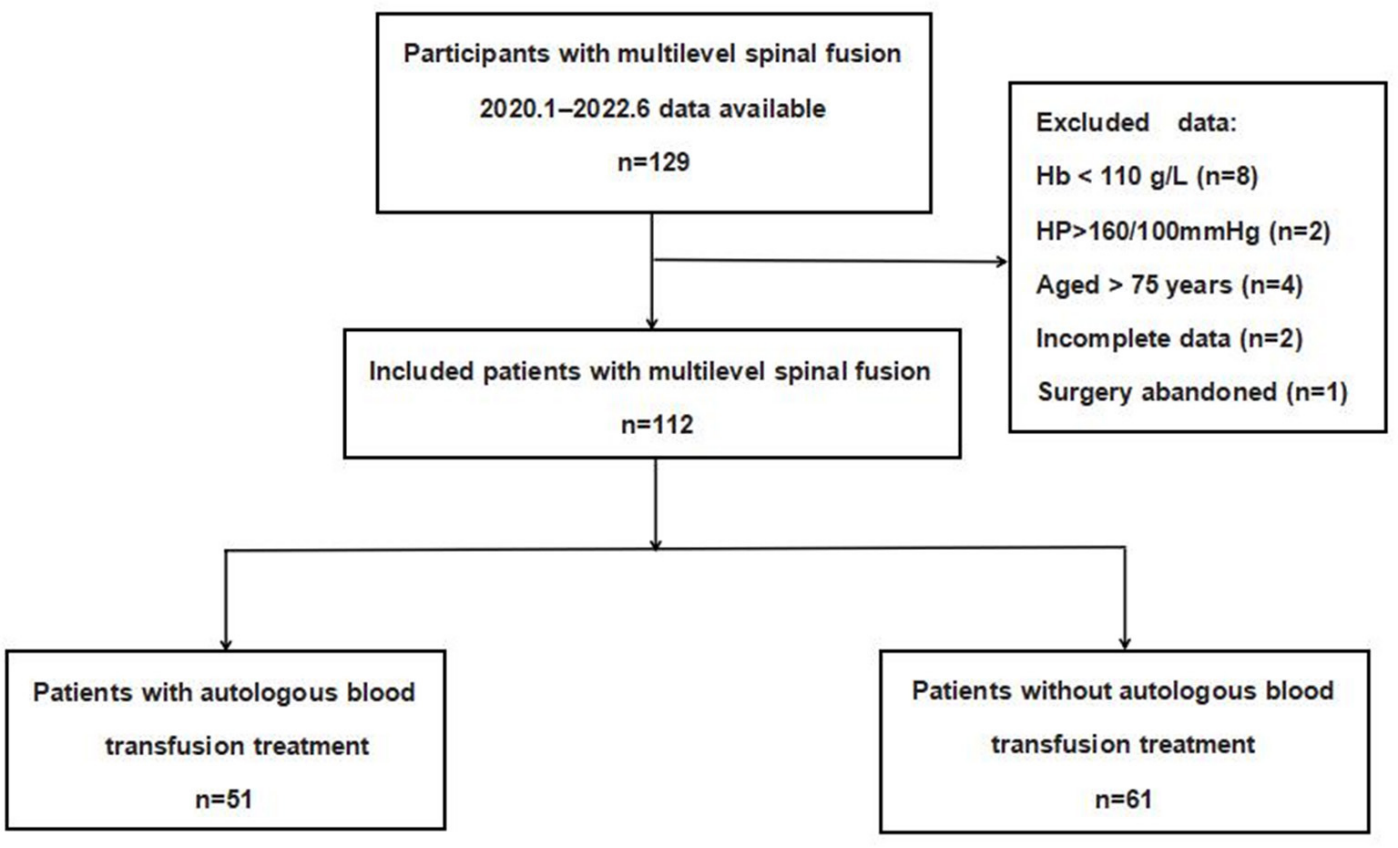
Flowchart of the study population.

Figure 1 depicts the flowchart of the study population. A total of 112 patients were finally statistically analyzed, of which 51 were included in the PARA group and 61 in the control group according to the presence or absence of PARA. A summary of the demographic and perioperative variables, including age, gender, BMI, ASA classification, number of fusion levels, and pathology type, is listed in Table 1. There was no statistically significant difference in demographic and clinical characteristics.

**Table 1:** Baseline demographic and clinical characteristics.

	**PARA group (*n =* 51)**	**Control group (*n =* 61)**	***P* value**
Age (Years)	57.22 ± 12.77	59.35 ± 13.01	0.168
Gender (Male/Female)	30/21	36/25	0.708
BMI	23.35 ± 1.33	23.10 ± 1.53	0.319
Blood volume (L)	4.20 ± 0.47	4.23 ± 0.44	0.331
ASA classification (I/II/III/IV)	0/12/36/3	0/14/45/2	0.793
Number of fusion levels	5.34 ± 3.68	5.51 ± 3.92	0.529
Pathology type (%)	
Fracture	6 (11.76)	8 (13.11)	1.000
Tumor	2 (3.92)	1 (1.64)	0.591
Degenerative	31 (60.78)	38 (62.30)	1.000
Deformity	10 (19.61)	11 (18.03)	0.643
tuberculosis	2 (3.92)	3 (4.91)	1.000

BMI, body mass index; ASA, American Society of Anesthesiologists.

### 3.2. Transfusion-related parameters and outcomes

The variables of transfusion-related parameters and outcomes between the PARA and control groups are listed in Table 2. Among all subjects, the intraoperative and perioperative allogeneic RBC transfusion amount was significantly higher in the control group than that in the PARA group (*p* < 0.001). Similarly, the intraoperative and perioperative allogeneic RBC transfusion ratio in the control group was also significantly higher than that in the PARA group (*p* < 0.001). Moreover, no significant differences were found in functional outcomes, including the VAS score (*p* = 0.159), ODI score (*p* = 0.214), JOA score (*p* = 0.752), and SF-36 score (*p* = 0.188) between the PARA and control groups.

**Table 2:** Transfusion-related parameters and outcomes.

	**PARA group (*n =* 51)**	**Control group (*n =* 61)**	***P-value***
Intraoperative allogeneic RBC transfusion volume (units)	0 (0,0)	4 (4,5)	< 0.001^***^
Intraoperative allogeneic RBC transfusion rate (%)	6 (11.76%)	59 (96.72%)	< 0.001^***^
Perioperative allogeneic RBC transfusion volume (units)	0 (0,0)	4 (4,5)	< 0.001^***^
Perioperative allogeneic RBC transfusion rate (%)	6 (11.76%)	59 (96.72%)	< 0.001^***^
**Functional Outcome**			
VAS score	1.27 ± 0.59	1.38 ± 0.68	0.159
ODI score	12.59 ± 1.26	12.38 ± 0.85	0.214
JOA score	27.98 ± 1.17	27.46 ± 1.21	0.752
SF-36 score	775.91 ± 55.48	746.54 ± 46.69	0.188

VAS, visual analog scale; ODI, Oswestry Disability Index; JOA, Japanese Orthopedic Association; SF-36, 36-Item Short-Form Health Status Survey. ^*^Statistically significant: *P* < 0.05, ^**^statistically significant: *P* < 0.01, and ^***^statistically significant: *P* < 0.001.

### 3.3. Hospitalization and transfusion Costs

The variables of hospitalization and transfusion costs between the PARA and control groups are listed in Table 3. Preoperative (*p* = 0.060) length of stay was similar in both groups. However, the postoperative (9.04 ± 3.21 vs. 11.05 ± 3.84; *p* = 0.004) and total length of stay (15.78 ± 3.79 vs. 17.36 ± 4.08; *p* = 0.038) in the PARA group were significantly less than those in the control group, respectively. Despite no difference in hospitalization cost (*p* = 0.737), the total blood transfusion cost in the PARA group was significantly lower, compared with the control group (*p* < 0.001).

**Table 3:** Hospitalization and transfusion costs.

	**PARA group (*n =* 51)**	**Control group (*n =* 61)**	***P-value***
Preoperative length of stay (day)	6.71 ± 1.12	6.31 ± 1.43	0.060
Postoperative length of stay (day)	9.04 ± 3.21	11.05 ± 3.84	0.004^**^
Total length of stay (day)	15.78 ± 3.79	17.36 ± 4.08	0.038^*^
Total blood transfusion costs (RMB)	0 (0,0)	880 (860, 1,330)	< 0.001^***^
Total Hospital costs (RMB)	68,424.50 ± 21,245.54	69,797.31 ± 18,665.51	0.737

^*^Statistically significant: *P* < 0.05, ^**^statistically significant: *P* < 0.01, and ^***^statistically significant: *P* < 0.001.

### 3.4. Safety evaluation of PARA

**Figure 2:**
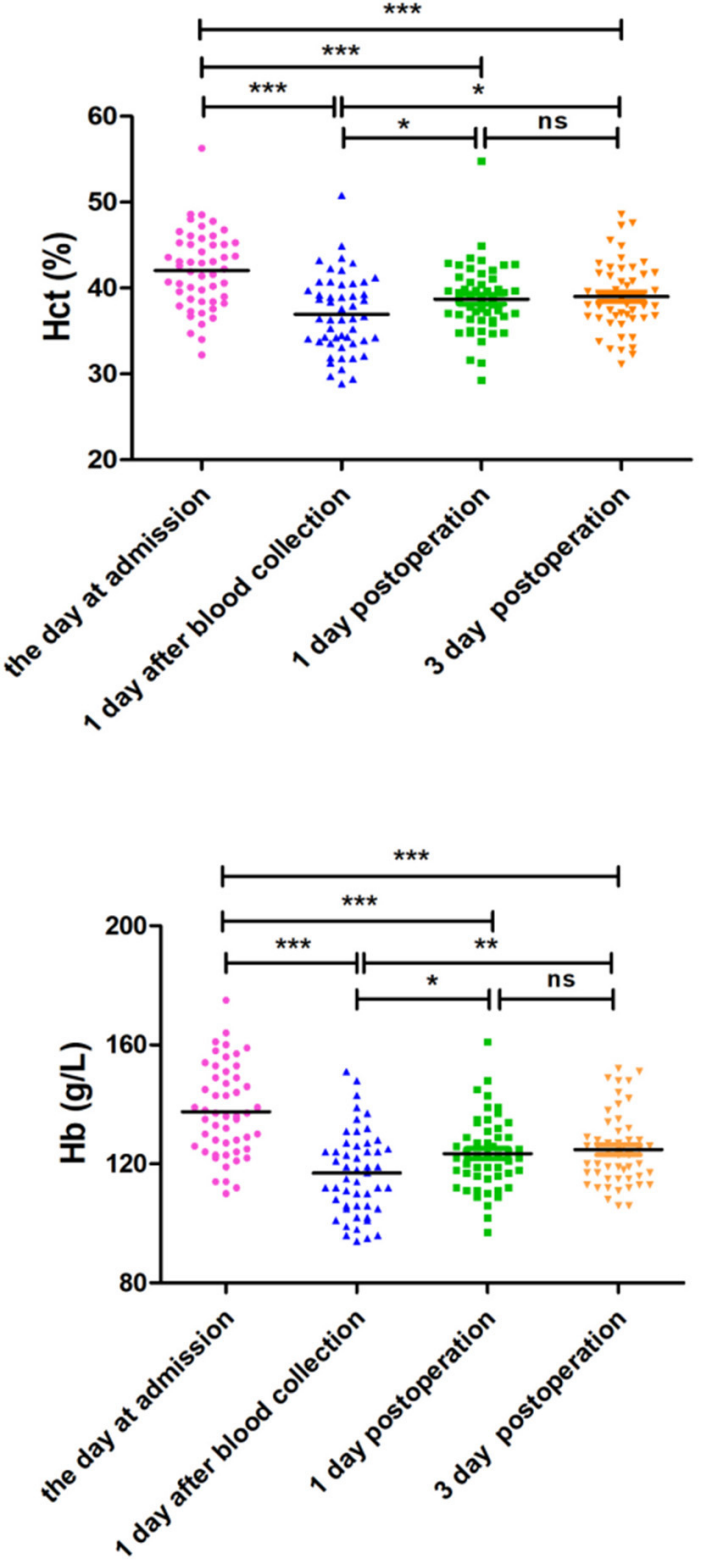
Changes in Hb and Hct levels after autologous blood collection.

The variables of safety evaluation of PARA between the PARA and control groups are listed in Table 4. The average volume of autologous blood preoperative collection was 4.39 ± 0.49 units in the PARA group. Although the Hb and Hct levels were decreased on postoperative day 1 in all patients, there were no significant differences in Hb and Hct levels between the two groups at admission, on postoperative day 1, and postoperative day 3, respectively (*p* > 0.05). For further evaluation for safety, the Hb and Hct levels at admission, 1 day after collection, postoperative day 1, and postoperative day 3 were compared in the PARA group. As expected, the Hb and Hct levels decreased sharply on the first day after collection within the control range, but the two variables could recover to normal levels in the PARA group on postoperative day 1 and day 3, despite significant differences when comparing the two groups, as shown in Figure 2, Supplementary Tables S1, S2 (*p* < 0.05).

**Table 4:** Changes in perioperative Hb and Hct.

	**PARA group (*n =* 51)**	**Control group (*n =* 61)**	***P value***
Volume of autologous blood collection (U)	4.39 ± 0.49	-	-
Hb level at admission (g/L)	137.55 ± 15.18	132.22 ± 14.45	0.105
Hct level at admission (%)	42.05 ± 4.52	40.79 ± 4.08	0.121
Hb level on the postoperative day 1 (g/L)	111.75 ± 14.41	115.86 ± 12.98	0.110
Hct level on the postoperative day 1 (%)	34.93 ± 4.88	35.86 ± 3.92	0.276
Hb level on the postoperative day 3 (g/L)	124.76 ± 12.13	127.00 ± 13.40	0.259
Hct level on the postoperative day 3 (%)	39.01 ± 4.01	38.86 ± 3.77	0.932

Hb, hemoglobin; Hct, hematocrit.

The variables of parameters related to transfusion complications between the PARA and control groups are listed in Table 5. During autologous blood collection, two cases of hypocalcemia reaction and one case of donation adverse reaction occurred in the PARA group. There was no significant difference in the incidence of postoperative complications and adverse blood transfusion reactions between the two groups (*p* > 0.05). However, the number of postoperative infections in the PARA group was significantly lower than that in the control group (*p* = 0.038).

**Table 5:** Parameters related to transfusion complications.

	**PARA group (*n =* 51)**	**Control group (*n =* 61)**	***P value***
**Adverse reactions of blood collection**			
Hypocalcemia reaction	2 (3.9%)	-	-
Donation adverse reaction	1 (1.9%)	-	-
**Postoperative complication**			
Infection	1 (2.0%)	8 (13.1%)	0.038^*^
Thrombosis	2 (3.9%)	3 (4.9%)	1.000
Mild anemia	6 (11.8%)	4 (6.6%)	0.508
**Transfusion reaction**			
Allergy	0 (0%)	2 (3.3%)	0.500
Nonhemolytic febrific reaction	0 (0%)	4 (6.6%)	0.124

^*^Statistically significant: *P* < 0.05.

## 4. Discussion

Although perioperative allogeneic blood transfusion was a major choice, the perioperative autologous blood preparation as a new concept of patient blood management (PBM), which has been revealed to be efficient in saving blood resources, reducing the amount of allogeneic blood transfusion, and ensuring the safety of transfusion, has been accepted and performed gradually (19). Among them, PAT has been applied in preoperative blood management when there is a high risk of intraoperative bleeding. However, some limitations of traditional PAT, including low blood-saving efficiency and quite a long time for mass autologous blood preparation, restrict the implementation and popularization (16, 20). The results of our study indicated that PARA was a novel method to ensure blood transfusion safety, which could solve the disadvantages of PAT. This was the first study about the efficacy and safety of PARA in patients undergoing multilevel spinal fusion surgery.

The rationale behind the PARA was based on the PAT and ANH. In brief, concentrated RBC was separated while maintaining the balance of blood volume in and out with normal saline, and the indications and contraindications of PARA were the same as PAT. However, there were certain advantages of PARA, compared with PAT (9, 20): (1) High efficiency of saving blood: 1U allogeneic RBC suspension used usually was obtained from 200 ml of whole blood. However, 400–500 ml of concentrated RBC (100 ml = 1 U) collected by PARA was transfused, which was equivalent to saving 800–1,000 ml of whole blood, compared with PAT. (2) Short preparation cycle: The preparation for 1,000 ml of autologous blood would take 3 weeks by PAT, as to the collection requirement (400 ml/week/person in China), but only about 30 min by PARA. (3) Component blood transfusion: The final product of PARA was concentrated RBC, rather than whole blood from PAT, which is more compatible with the clinical requirements of component transfusion. (4) After the collection process, the remaining whole blood and plasma left in the machine were transfused back into the patient automatically, so the coagulation factors and platelets were not lost, compared with PAT. (5) Quick recovery: The Hb and Hct levels of patients in the PARA group could return to normal levels after concentrated RBC collection within approximately 5 days. The probable explanations are that preoperative blood donations could stimulate bone marrow cell proliferation and stimulate erythrocyte regeneration, but the mechanism of that is unclear and needs to be revealed in further study.

Hospital cost and length of stay are important assessment indicators worldwide due to the medical insurance systems, especially in European and North American countries (21, 22). To our knowledge, the hospital cost could be decreased by autologous blood transfusion (9, 23), especially the transfusion cost, which was not in accordance with our results completely. Although the perioperative transfusion cost in the PARA group was significantly less than those in the control group, the total hospital cost in both groups was still similar. The reason was a large amount of intraoperative allogeneic RBC transfusion in the control group compared with the PARA group. Moreover, due to the large total Hospital cost, the decline in transfusion cost was insufficient to cause statistical differences in this index.

It was notable that there was no difference in the preoperative length of stay between the two groups, while the postoperative and total length of stay in the PARA group were significantly shorter than those in the control group. Several studies have indicated that intraoperative allogeneic RBC transfusion may impair patients' immune function and cause infection, which is not conducive to postoperative outcomes (9). Thus, the possible explanation for our result is that allogeneic blood transfusion could upregulate the expression of immunosuppressive prostaglandin, inhibit the bactericidal ability of immune cells, reduce lymphocytes, prevent lymphocyte proliferation, and decrease the activity of natural killer cells, resulting in an increased risk of perioperative infection (24, 25). In this study, compared with the PARA group, the higher postoperative infection may be a major reason for prolonging postoperative hospital stay in the control group. Thus, it is necessary to reduce allogeneic blood transfusion in patients undergoing multilevel spinal fusion surgery during preoperative preparation.

The safety of this technique is another important evaluation index in this study, as reflected in the following aspects: (1) The blood volume in and out was maintained balance during the whole process to avoid hypotension by PAT. (2) The target value of Hct was set as 0.3, referring to AHN, which could promote the right shift of the oxygen dissociation curve, increase the oxygen uptake capacity of the tissue, improve microcirculation, and reduce ischemia–reperfusion injury (17, 26, 27). However, the upper limit of collection volume was set at 500 ml (10% of blood volume) in order to avoid anemia after blood collection caused by excessively concentrated RBC loss. Although decreasing sharply after preoperative blood collection in the PARA group, there was no significant difference in perioperative Hb and Hct levels between the two groups. The probable reason may be that preoperative blood collection could stimulate the proliferation of bone marrow hematopoietic cells. Therefore, the upper limit of the collection volume should be explored in a future study. (3) Only two cases of hypocalcemia and one case of blood donation reaction occur during blood collection, which were solved immediately by doctors. Therefore, PARA was a safe method with rare adverse for preoperative autologous blood preparation.

Moreover, PARA has a good application prospect and is suitable for major selective surgeries such as cardiothoracic, hepatologic, urinary, and even gynecology and obstetrics surgery. More blood resources could be saved effectively by PARA. In our opinion, some principles should be followed when PARA is performed. (1) Patients should be screened strictly according to inclusion criteria. (2) In some special patients undergoing cardiothoracic and obstetrics surgery, the volume of PARA should be reduced to 300–400 ml. (3) For safety, monitoring using an ECG monitor must be carried out during the whole process.

There were also certain limitations in this study. First, this was a single-center retrospective study, which might lead to inherent biases, and these results require further external validation. Prospective randomized controlled studies with large cohorts are required owing to the limited sample size. Second, the comparison of efficacy between PARA and other autologous blood transfusion techniques was not performed simultaneously in this study. Thus, involving this comparison should also be considered in our subsequent study design. Third, the mechanism of quick recovery after autologous RBC collection was unclear, so further research is necessary to illustrate this question.

## 5. Conclusion

In conclusion, PARA is probably a novel, safe, and highly efficient technique for mass autologous blood preparation with a quite short preparation time. This method could significantly save allogeneic RBC and reduce the amount of allogeneic blood transfusion and length of stay, which could provide a theoretical basis for using the technique in clinical practice.

## Data availability statement

The original contributions presented in the study are included in the article/Supplementary material, further inquiries can be directed to the corresponding authors.

## Ethics statement

This study was approved by the Ethics Committee of the First Affiliated Hospital of Chongqing Medical University (Approval No: 2020-232). The patients/participants provided their written informed consent to participate in this study.

## Author contributions

X-PX, X-LY, and JH designed this study and wrote the manuscript. X-PX, W-JQ, Z-BY, and J-LS performed the experiments. X-PX, W-JQ, Z-BY, Z-MH, and X-LY collected and provided the sample for this study. All authors discussed the results and contributed to the final version of the manuscript.
