# Supplementary material for: Clinical application of a modified predeposit autologous red blood cell apheresis in multistage spinal fusion: a single-center retrospective study
Source: Front Med (Lausanne). 2023 May 15;10:1149093. doi: 10.3389/fmed.2023.1149093 (PMC10225601; doi:10.3389/fmed.2023.1149093)
Supplement: Supplementary file 1 [file Data_Sheet_1.doc]

**Table S1. Changes of Hb and Hct in the PARA Group**

|  | **Hb level（g/L）** | **Hct level (%)** |
| --- | --- | --- |
| The day at admission | 137.55±15.18 | 42.05±4.52 |
| 1 day after collection | 116.90±14.07 | 36.94±4.49 |
| The postoperative day 1 | 123.47±12.23 | 38.71±4.05 |
| The postoperative day 3 | 124.76±12.13 | 39.01±4.01 |

**Table S2. Comparison of Hb and Hct at different time in the PARA Group**

|  | ***P value* of Hb level** | ***P value* of Hct level** |
| --- | --- | --- |
| Admission *VS* 1 day after collection | <0.001*** | <0.001*** |
| Admission *VS* Postoperative day 1 | <0.001*** | <0.001*** |
| Admission *VS* Postoperative day 3 | <0.001*** | <0.001*** |
| 1 day after collection *VS* Postoperative day 1 | 0.029* | 0.017* |
| 1 day after collection *VS* Postoperative day 3 | 0.02* | 0.004** |
| Postoperative day 1 *VS* Postoperative day 3 | 0.219 | 0.575 |

*Statistically significant: *P*<0.05, **Statistically significant: *P*<0.01, ****Statistically significant: *P*<0.001
